# Patient-Level Effectiveness Prediction Modeling for Glioblastoma Using Classification Trees

**DOI:** 10.3389/fphar.2019.01665

**Published:** 2020-01-31

**Authors:** Tine Geldof, Nancy Van Damme, Isabelle Huys, Walter Van Dyck

**Affiliations:** ^1^ Healthcare Management Centre, Vlerick Business School, Ghent, Belgium; ^2^ Department of Pharmaceutical and Pharmacological Sciences, Research Centre for Pharmaceutical Care and Pharmaco-economics, KU Leuven, Leuven, Belgium; ^3^ Belgian Cancer Registry, Brussels, Belgium

**Keywords:** real world evidence, oncology, exploratory study, propensity score modeling, decision tree, machine learning

## Abstract

**Objectives:**

Little research has been done in pharmacoepidemiology on the use of machine learning for exploring medicinal treatment effectiveness in oncology. Therefore, the aim of this study was to explore the added value of machine learning methods to investigate individual treatment responses for glioblastoma patients treated with temozolomide.

**Methods:**

Based on a retrospective observational registry covering 3090 patients with glioblastoma treated with temozolomide, we proposed the use of a two-step iterative exploratory learning process consisting of an initialization phase and a machine learning phase. For initialization, we defined a binary response variable as the target label using one-by-one nearest neighbor propensity score matching. Secondly, a classification tree algorithm was trained and validated for dividing individual patients into treatment response and non-response groups. Theorizing about treatment response was then done by evaluating the tree performance.

**Results:**

The classification tree model has an area under the curve (AUC) classification performance of 67% corresponding to a sensitivity of 0.69 and a specificity of 0.51. This result in predicting patient-level response was slightly better than the logistic regression model featuring an AUC of 64% (0.63 sensitivity and 0.54 specificity). The tree confirms confounding by age and discovers further age-related stratification with chemotherapy-treatment dependency, both not revealed in preceding clinical studies. The model lacked genetic information confounding treatment response.

**Conclusions:**

A classification tree was found to be suitable for understanding patient-level effectiveness for this glioblastoma–temozolomide case because of its high interpretability and capability to deal with covariate interdependencies, essential in a real-world environment. Possible improvements in the model’s classification can be achieved by including genetic information and collecting primary data on treatment response. The model can be valuable in clinical practice for predicting personal treatment pathways.

## Introduction

Glioblastoma is one of the most common and aggressive brain tumors in adults, with a median survival of less than one year from the time of diagnosis. Apart from the current standard of care treatment based on surgical resection and post-operative radiotherapy, there is only one medicinal product available for the treatment of glioblastoma patients. This temozolomide intervention has been shown to be efficacious in prolonging survival in Randomized Controlled Trials (RCTs) (Stupp et al., 2005; Stupp et al., 2009).

However, specific details on the mechanisms that drive individual response to temozolomide treatment in clinical practice, or on the drivers of real-world patient-level treatment effectiveness, are unknown (van Genugten et al., 2010; Eichler et al., 2011; Liu et al., 2016). To study these personal responses, traditional cohort-oriented methods, such as the Kaplan-Meier survival techniques currently used in pharmacoepidemiology (Strom and Kimmel, 2006) for investigating real-world evidence (RWE) data, have shown to be inadequate because of their difficulties to cope with heterogeneous patient populations; their restrictive assumptions regarding linear relationships among variables; their inability to provide patient-level predictions; and their inability to infer causality (Ankarfeldt et al., 2017; Arora et al., 2019).

For example, Kaplan-Meier methods provide (sub)population-level results, that is, they return the average or median treatment effect rather than patient-level results. Other statistical methods commonly used in the domain of medicine, such as logistic regression models, have hitherto focused mainly on investigating survival probability and their associated confounding factors when used in pharmacoepidemiology, as opposed to treatment effectiveness (Burke et al., 1995).

While currently primarily investigated for their application in drug discovery and development (Vamathevan et al., 2019), Onukwugha et al. (2017) suggested machine learning to be a valuable tool in pharmacoepidemiology as well aiming at studying this personal treatment’s effectiveness (Onukwugha et al., 2017). Specifically, conducting exploratory treatment effectiveness studies using machine learning generates new knowledge on whether and how the treatment works in its specific real-world population and health care system context by accurately making individual predictions (Onukwugha et al., 2017; Berger et al., 2017; Puranam et al., 2018). These methods are increasingly being used by oncologists for cancer detection and prediction of risks, cancer recurrence, and survival (Lavrac, 1999; Kononenko and Kukar, 2001). Henceforward, machine learning develops as an alternative for traditional survival methods because it can be used for hypotheses generation on patient-level treatment effects in heterogenetic real-word patient populations, among others, through causal assessments (Vamathevan et al., 2019; Lavrac, 1999; Kononenko and Kukar, 2001; Cruz and Wishart, 2006; Onukwugha et al., 2017; Berger et al., 2017; Puranam et al., 2018). However, only little research has been done so far to explore the value of machine learning in pharmacoepidemiology (Crown, 2015).

In this paper, we present information-based machine learning methods – decision tree-based classification or classification trees (CT)—for use in a two-step iterative exploratory learning process to investigate the stratification factors of individual treatment response to temozolomide in glioblastoma patients using observational data. The well-known CT technique can then be used for patient-level effectiveness predictions of temozolomide.

## Materials And Methods

To investigate the effects of real-world data (RWD) covariates on real-world treatment response on a patient-level basis and to be able to identify confounding factors influencing real-world treatment response, the methods that are used should allow for product performance-based data labeling if no primary data are available on real performance per patient. Hence, these models should use patient-level information and be able to handle personal treatment paths and/or genomic information. In this section, we will first describe the data collection process and provide a definition of the product’s performance used to annotate the data set. Next, we will describe the classification models and exploratory learning process used for theorizing about personal treatment effectiveness.

### Data Setting

In this study, data were extracted from the Belgian Cancer Registry (BCR), including 4587 patients with glioblastoma (ICD-10 code C71.0-C71.9) diagnosed between 2004 and 2012, and vital status information updated until January 1, 2015. Variables for this study were taken from the full standard set of variables nationally collected by the BCR—including patient and tumor characteristics—and Inter Mutualistic Agency (IMA), including reimbursed therapeutic acts consisting of medical acts and medications administrated in hospitals and handed out in pharmacies. These variables were further limited by BCR oncologists for their potential relevance in the analysis.

The index date, or date of incidence of glioblastoma, was defined as the date of first microscopic confirmation of malignancy, first hospitalization for the cancer, first consultation for the malignancy, first clinical or technical diagnosis, start of treatment, or date of death, whichever date came first. Patients with incidence dates that were the same as the date of death as well as patients without a social security identification number were excluded.

Temozolomide therapy relevant for the treatment of glioblastoma was extracted from the IMA data set based on the medicines’ anatomical therapeutic chemical (ATC) code (L01AX03) and treatment start data within −1 to 9 months from the date of incidence. Other chemotherapeutic interventions with possible interactive effects were extracted from the IMA data set based on the ATC code for chemotherapy (L01), starting −1 month from the date of incidence. Information on radiotherapeutic (RT) interventions, biopsy, and surgical resection were extracted from the IMA data set by BCR51 oncologists based on the relevant nomenclature codes used.

The final data set consisted of (a) the patient’s overall survival (OS) period, a continuous variable calculated as the difference between the date of death or last confirmation that the patient was alive and date of incidence; (b) treatment path, that is, binary variables indicating biopsy and/or surgical resection and RT, and chemotherapeutic treatment; (c) five discrete covariates (age, tumor differentiation grade, topography, total number of tumors, and World Health Organization [WHO] performance score at diagnosis and recursive partitioning analysis [RPA] class), one binary covariate (sex), and one categorical covariate (tumor topography, specifying the location in the brain), confounding both the patient’s OS and treatment path; and (d) OS binary observation status specifying whether the survival was censored, that is, whether the follow-up time was too short to observe the date of death. The final RWD set consisted of 4528 patients, of which 3090 treated with temozolomide (**Table 1**).

**Table 1:** Main characteristics of the real-world study population.

	Real-World	
	Control group (n = 1438)	Treated group (n = 3090)
**Age**		
Range (median)	0–94 (74)	5–98 (61)
no. (%) < 50	98 (42%)	582 (19%)
no. (%) >= 50	1,340 (58%)	2,508 (81%)
**Sex – no. (%)**		
Male	814 (57%)	1,847 (60%)
Female	624 (43%)	1243 (40%)
**WHO performance status—n (%)**		
0—asymptomatic	253 (18%)	415 (13%)
1—symptomatic but completely ambulatory	850 (59%)	2265 (73%)
2—symptomatic, up and about >50% walking hours	197 (14%)	313 (10%)
3—symptomatic, confined to bed/chair > 50% walking	84 (6%)	61 (2%)
hours	54 (4%)	36 (1%)
4—completely disabled; totally confined to bed/chair		
**RPA—n (%)**		
Class III†	43 (3%)	162 (5%)
Class IV‡	789 (55%)	2,419 (78%)
Class V §	606 (42%)	509 (16%)
**Surgical procedure (biopsy/debulking)—n (%)**		
No	169 (12%)	23 (1%)
Yes	1,269 (88%)	3,067 (99%)
**Radiotherapy treatment—n (%)**		
No	899 (63%)	130 (4%)
Yes	539 (37%)	2,960 (96%)
**Chemotherapy treatment—n (%)**		
No	1,342 (93%)	2,277 (74%)
Yes	96 (7%)	813 (26%)
**Time from diagnosis to radiotherapy:** range (median)	377.0–256.3 (Arora et al., 2019)	−313.6 to 186.9 (Vamathevan et al., 2019)
**Time from diagnosis to chemotherapy:** range (median)	−4.0 to 190.0 (Stupp et al., 2005)	−4.3 to 389.7 (Burke et al., 1995)

Patients were categorized according to recursive partitioning analysis (RPA) classes: †Age < 50 years and World Health Organization (WHO) status 0. ‡ Age < 50 years and WHO status > 0 or age ≥ 50 years and surgical resection. §Age ≥ 50 years and no surgical resection

### Definitions

Because no primary data on treatment response was available for temozolomide, initialization was needed to label the data. For this purpose, a binary dependent variable with variables 1 and 0 representing individual-treatment response and non-response, respectively, was created based on the patients’ gain in OS, that is, the number of months the patient gained in survival when being assigned to the temozolomide treatment. Here, OS was used as the main indicator of the treatment effect because this was the RCT’s primary endpoint. Patients’ gain in OS was calculated using nearest neighbor propensity score (PS) matching, a method commonly used on RWD to mitigate bias induced by the non-random assignment of treatments. Hence, let *T* and *C* be the set of treated (Z = 1) and control (Z = 0) patients, respectively. The PS = Pr(*Z*_*i*_ = 1|*X*_*i*_) is defined as the probability of being assigned to the treatment of consideration conditional on the observed covariates X. Its value is estimated using a logit model (Rosenbaum and Rubin, 1983; Rosenbaum and Rubin, 1984) with the selected covariates X being the observed variables which significantly affect the survival time, because this variable selection approach is associated with better PS estimations (see supplementary materials for more details) (Austin et al., 2007). Following this nearest neighbor PS technique, each temozolomide-treated patient is matched to k control patients based on the smallest difference in estimated PSs, that is, *i* ∈ *T* and *j* ∈ *C* are matched if *dist* $\left(PS_{i}^{T},PS_{j}^{C}\right)$ is minimal (Rosenbaum and Rubin, 1983; Rosenbaum and Rubin, 1984).

Here, we chose to set k equal to 10, given a set of 1438 control patients, to not average out possible covariate effects. This nearest neighbor PS matching algorithm was performed with the “MatchIt” 125 package within R (Ho et al., 2011).

Further, let *Y* *_T_* = *OS* *_T_* and *Y* *_C_* = *OS* *^C^* be the observed continuous outcomes of the treated and control units, respectively. Denote by *C* (*i*) the set of k control patients *j* ∈ *C* matched to the treated patient *i* ∈ *T*. Define the weights *w*_*ij*_ = 1/*k* if *j* ∈ (*i*) and *w*_*ij*_ = 0 otherwise. From the formula for the average treatment effect (Ho et al., 2011), we defined the treated patient’s survival gain (SG): $SG_{i}=OS_{i}^{T}-\sum_{j\in C(i)}w_{ij}OS_{j}^{C}$. Following the guidelines of the European Society for Medical Oncology (ESMO) andMagnitude of Clinical Benefit scale (MCBS) and with the aim of maximizing treatment response rate(TRR) (Becker and Ichino, 2002), patients were labeled with “response” whenever their SG was longer than the threshold *λ* equal to one month (Cherny et al., 2015).

### Classification Model

We used classification techniques within machine learning to divide individual patients into treatment response and non-response groups, with the purpose to fully understand individual treatment response to temozolomide. For exploratory reasons, we used a CT to extract patterns from the data. CTs are highly interpretable and intuitive as well as well attuned to coping with missing data and heterogeneous data types (Kelleher et al., 2015). While recursively creating branches for different covariate values, ordered in function of their classification error minimization power, the CT algorithm (for details see **Supplementary Material**) gradually improves prediction accuracy. Missing data is handled by classifying these observations in branches based on surrogate variables, predicting the most likely missing variable value.

As pointed out by Puranam et al. (2018), we believe that our sample size of 3090 temozolomide-treated patients was sufficiently large to extract valuable evidence (Shaikhina et al., 2017). Although identification of the best classification model was not the main purpose of this research, we did compare this technique with a logistic regression model, one of the most commonly used statistical classification methods in the medicinal literature (Kononenko and Kukar, 2001).

The set of treated patients T was divided into a training set, comprising 80% (2472 units) of the temozolomide-treated patients sampled at random, and a test set, comprising the remaining 20% (618 units). The CT algorithm was trained and validated using 10-fold cross validation to obtain the most generalizable model using the “rpart” package within R, which implements the Classification and Regression Tree (CART) algorithm described by Breiman et al. (1984). Given that the difference between our defined binary response and predicted response by the classification model can be described by a confusion matrix, we can define the following properties: the number of true positives (TP), true negatives (TN), false positives (FP), and false negatives (FN). From these properties, the true positive rate (TPR) and the true negative rate (TNR) are defined as *TPR* = *TP*/(*TP* + *FN*) and *FPR* = *FP*/(*TN* + *FP*), respectively. The CT and logistic regression model performance were then evaluated by calculating the area under the curve (AUC) of the receiver operating characteristic (ROC) curve, mapping the models’ sensitivity and specificity measured by the TPR and 1 –TNR, respectively (Fawcett, 2006; Cherny et al., 2015). The AUC and ROC curves were computed using the “pROC” package within R (Robin et al., 2011).

### Iterative Exploratory Learning Process

**Figure 1:**
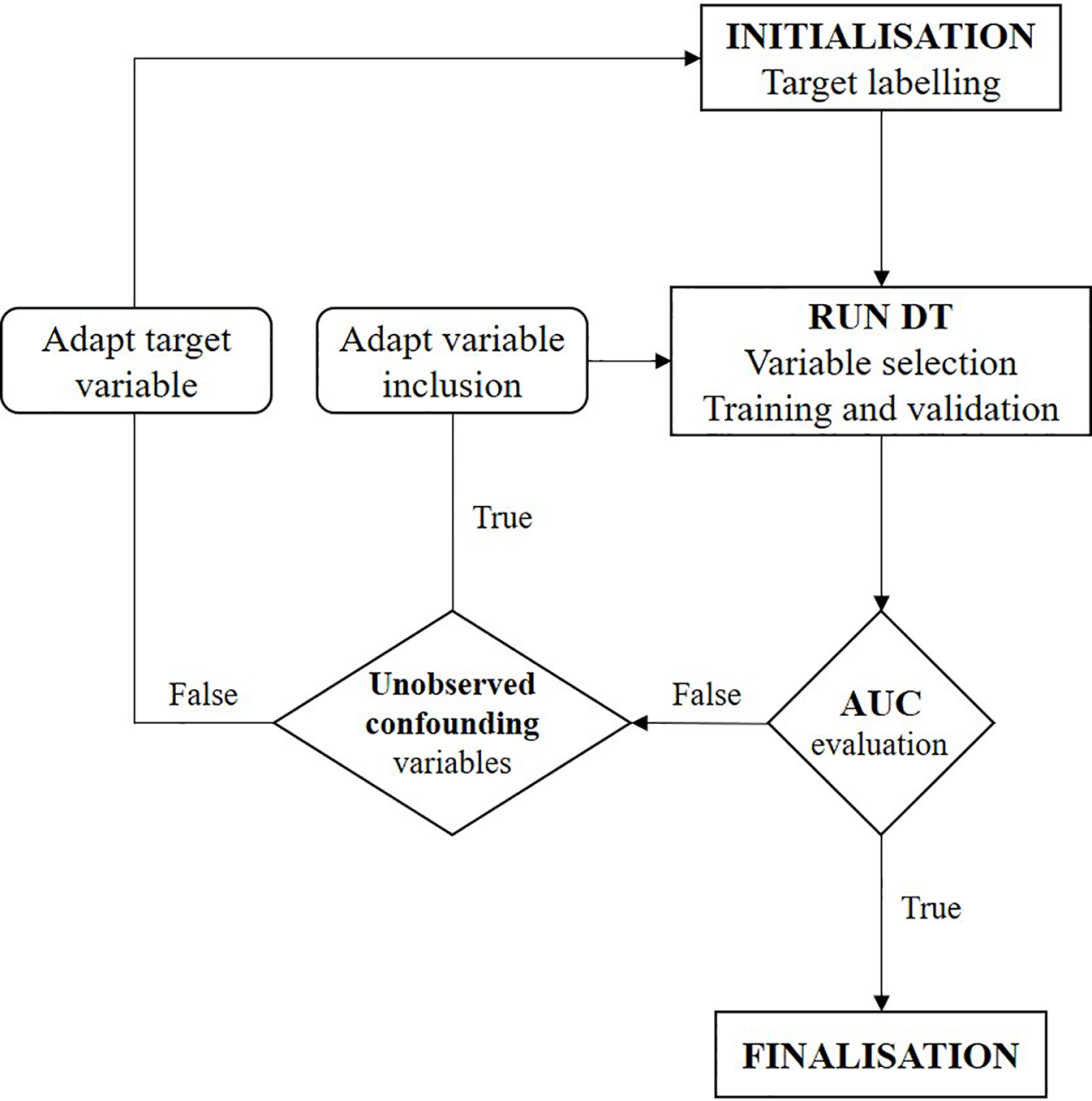
Flowchart of two-step iterative exploratory learning process. The model is iterated until the area under the receiver operating characteristic curve (AUC) is satisfactory, i.e. until the highest achievable AUC in practice is found. Unobserved confounding variables are (unknown) variables currently not captured in real-world situations. (AUC, area under the receiver operating characteristic curve; CT, decision tree).

The focus of this study was on investigating the confounding factors and causal effects of individual treatment response to temozolomide. As classification methods within machine learning identify correlations but cannot by themselves reach causal inference (Puranam et al., 2018), further interpretation of the CT is required. We conducted a two-step iterative exploratory learning process, as depicted in **Figure 1**, which aids inductive theory building. This learning process consisted of the evaluation of (i) possible unobserved confounding variables, for example through expert consultation, and (ii) the redefinition of response as a target feature when not available as primary data, by changing TRR assumptions and/or using different response-identification algorithms. Iteration ended when no further improvements were obtained, giving the model’s optimal AUC achievable in practice (see Appendix for pseudo-code).

## Results

First, we will show results for the data labeling process for patients treated with temozolomide. Thereafter, the outcome of the trained and validated CT is given and evaluated. The training set for the CT model consisted out of 2472 temozolomide-treated patients. These CT results are finally compared to the results of the logistic regression model.

### Initialization: Binary Response Labeling

The observed covariates significantly affecting the survival time of temozolomide-treated patients included patients’ age, RT, and chemotherapeutic treatment (p-value < 0.001), and WHO performance score (p-value < 0.01) (see supplementary materials for more details). Nearest neighbor PS matching based on these covariates resulted in 1063 control units matched once or multiple times to one treated unit. Following the ESMO-MCBS (Cherny et al., 2015), we obtained a TRR of 52%, meaning 1,607 of 3,090 temozolomide-treated patients showed SG > 1 month.

### Classification Results

**Figure 2:**
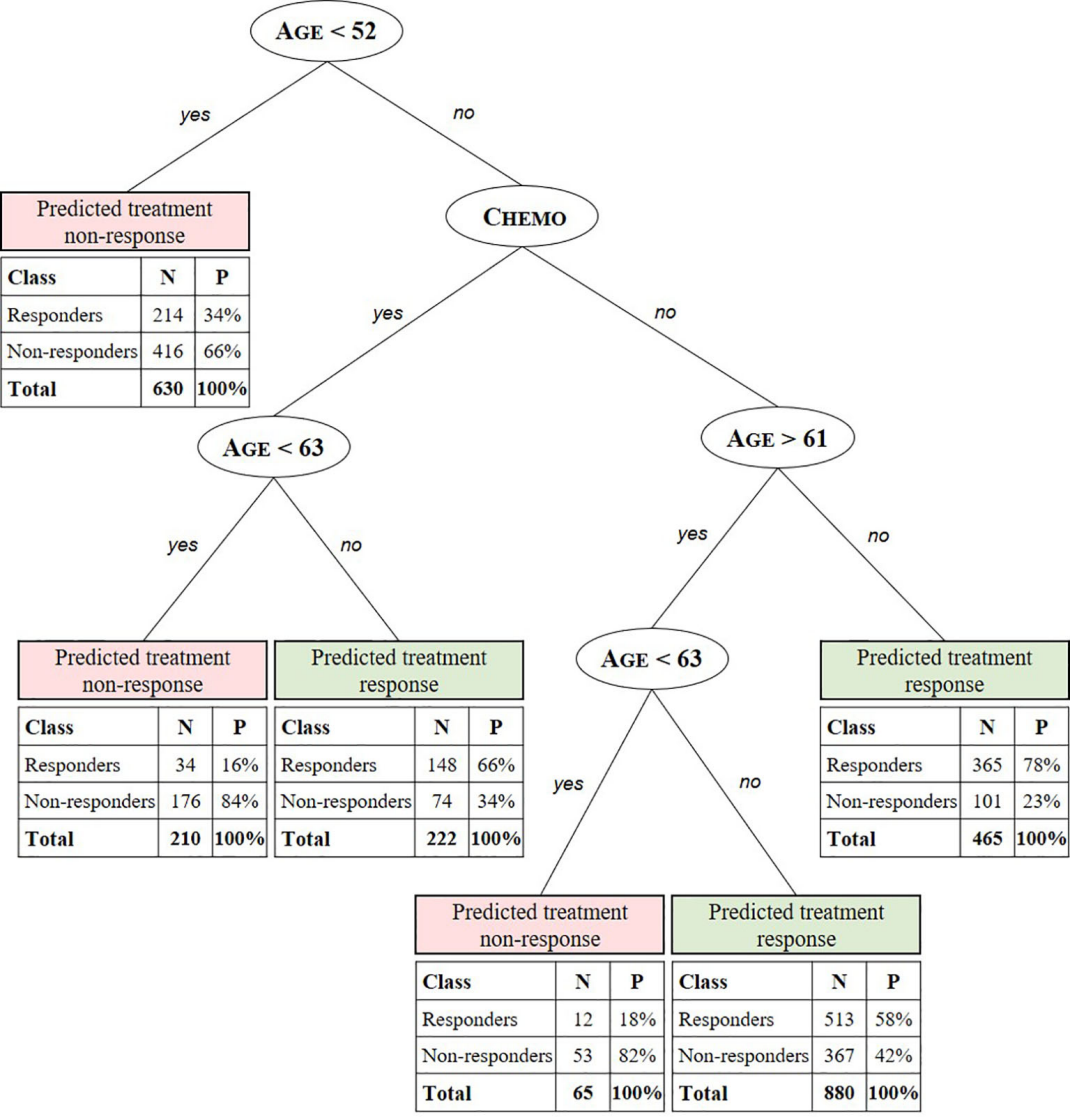
Summary predictive classification tree model after training and validation. Predicted stratification variables for TMZ in glioblastoma include age, RPA class, and chemotherapeutic (Chemo) and radiotherapeutic (RT) patient status. For each stratified patient class a confusion matrix indicates the number (N) and percentage (P) of treated patients from the test set for which the CT predicts treatment response correctly (responders to predicted treatment response and non-responders to predicted non-response) with respect to the labeled SG value. E.g. the CT model predicts the class of patients aged 52 to 61 years and >63 years not receiving concomitant or adjuvant chemotherapy to respond to the treatment with a true positive (TP) probability of 58%. For this class, with patients aged < 63 years 82% are correctly predicted (true negative [TN]) not to respond.

**Figure 3:**
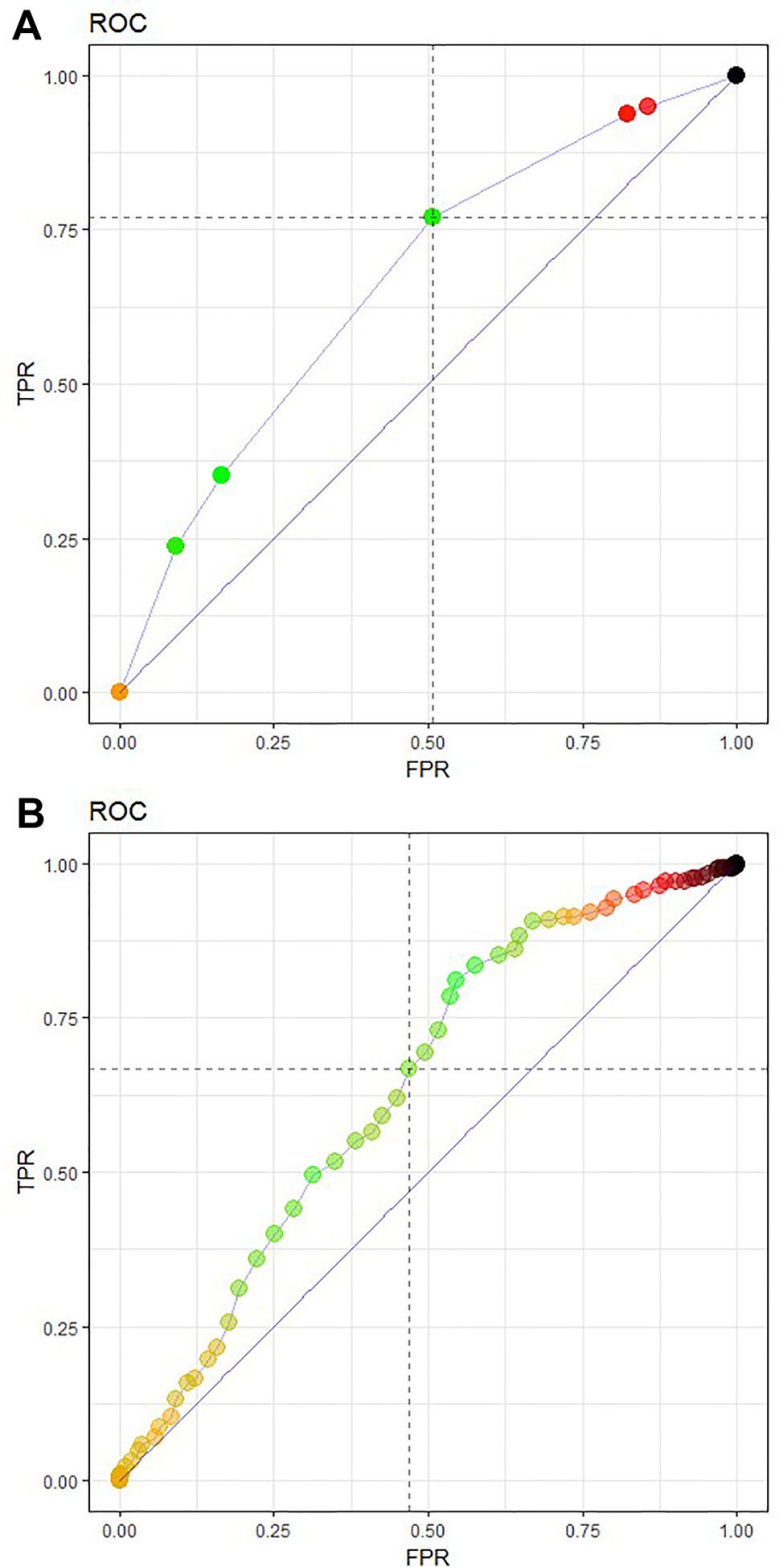
Receiver operating characteristic (ROC) curve featuring model performance evaluation as an area under the curve (AUC), sensitivity TPR and FPR or (1-specificity) for **(A)** the CT prediction model 3 and **(B)** a logistic regression model of the test data set. The CT model **(A)** featured an AUC of 0.6650, a sensitivity of 0.6850, and a specificity of 0.5114, The logistic regression model **(B)** achieved a slightly lower AUC of 0.6357 with a sensitivity of 0.6337 and specificity of 0.5420.

The CART algorithm showed a maximal decrease in classification error when first dividing the treated patients according to their age (**Figure 2**, see supplementary materials for more details). Another covariate stratifying the training set included patients’ chemotherapeutic treatment path, but such covariate interdependencies are currently not analyzed in RCT and treatment effectiveness studies (Stupp et al., 2005; Strom and Kimmel, 2006; Stupp et al., 2009; van Genugten et al., 2010). CT performance evaluation of the test data set resulted in an AUC of 0.6650 (**Figure 3A**). Compared to a model that is no better than a random classifier, featuring an AUC of 0.50, the CT performed better than chance but still showed poor prediction skills. Associated with this AUC was a sensitivity of 0.6850, meaning that 31% of patients who would benefit from the treatment were not recognized by the model, and a specificity of 0.5114, meaning that 49% of patients who would not benefit from the treatment were predicted to benefit by the model.

The logistic regression model achieved a slightly lower AUC of 0.6357 with a sensitivity of 0.6337 and specificity of 0.5420 (**Figure 3B**). Although they showed a better specificity than the CT, the results of the logistic regression model are still far too low.

### Iterative Exploratory Learning Process

With an AUC of 66.50% and 63.57% for the CT and logistic model, respectively, further interpretation of the model was done to obtain a higher sensitivity and lower specificity. In this temozolomide case, two learning steps were followed depicted in **Figure 1**: (i) theorization about possible unobserved confounding variables and (ii) redefinition of treatment response as a target feature. In the first case, a low AUC, which is associated with many misclassifications (false responders and non-responders), can result from the problem of spuriousness, suggesting that there may be some important confounding variables that were omitted from the data set, that were not collected in the data source, or that were just unknown (i.e., not known from any translational research). As an example, from our case, the BCR does not dispose of genetic information such as the methylation of the promoter for the gene encoding O-6-methylguanine-DNA methyltransferase. However, based on clinical research literature, this appears to be associated with a higher survival benefit (Stupp et al., 2005; Stupp et al., 2009).

In the second case, one can modify the TRR definition. In our case, for example, modifying the threshold to 3 months (giving a TRR of 43%) in the algorithm led to a CT with a different structure and lower AUC of 0.6005 (see supplementary materials). Again, age and status of chemotherapeutic treatment were shown to be the main classification variables.

## Discussion

Although the prediction structure induced by RWD confirms the importance of patient age, which was previously used as a stratification variable during RCT, the CT based on observational data reveals extra interdependencies of chemotherapy as a co-treatment effect, which was not found in preceding RCT-based studies. Such variable interdependencies cannot be investigated through current pharmacoepidemiology methods, including Kaplan Meier survival analysis techniques. In the following sections, we will discuss the causality assessment to generate hypotheses about personal treatment effectiveness and show the significance of this method. Next, we will discuss some limitations of the proposed method as well as possible issues with the data.

### Hypotheses Generation Through Exploratory Learning

Our CT model had an AUC of 67% with an associated sensitivity equal to 0.69 and specificity equal to 0.51. In the case of cancer treatments, a low specificity is undesirable because the treatment of false positives can be dangerous for the patient, depriving him or her of correct treatment, and can also be very costly, considering the high oncology drug prices during health care budget austerity. Therefore, theorizing about personal treatment effectiveness was done following an iterative learning process. A starting point for the first learning step of the CT was to explain why false responders and non-responders were observed in the various groups because this could suggest that there are some essential variables not being collected in RWD, such as genomic information, or other unidentified factors confounding RWE that are not detected by cohort-oriented methods used in current efficacy and effectiveness studies. Mitigating this problem of spuriousness may be essential to avoid wrong causal conclusions. Thus, including known or yet unknown unobserved (depending on data set used) confounding variables, for example through expert-consolations or conducting translational research, may lead to a subsequent CART search to induce a CT with better prediction accuracy and possibly a higher specificity.

In the second learning step, one can experimentally modify the TRR definition (under the guidance of experts) and/or method. Ideally, this can be done by collecting a treatment response identifier as primary data from the data source, such as information on tumor growth. Here, the TRR was based on PS matching and a non-variable SG threshold of 1 month. Depending on the extent of the phenotype (e.g. blood pressure) and genotype (e.g. mutations) variable collection in RWD sources, advanced TRR identification algorithms can greatly improve the labeling.

### Patient-Level Effectiveness Prediction

We found a combination of age and chemotherapeutic treatment status to be the main stratification factors of real-world personal treatment response to temozolomide in glioblastoma. Additionally, further specifications of these factors not found in preceding RCT-based studies were discovered. For example, the CT predicts positive response to the treatment for patients being assigned to chemotherapeutic treatment and being older than 63 years with a probability of 66%. Additionally, patients aged 52 to 61 years and >63 years not receiving concomitant or adjuvant chemotherapy are predicted to respond to the treatment with a probability of 58%. Using the iterative learning process described in Hypotheses Generation Through Exploratory Learning section, a higher AUC and hence better predictions could be obtained when (un)known stratification factors are identified and included. As an example, in our case, the BCR does not yet dispose of genetic information, such as the methylated promoter for the gene encoding O-6-methylguanine-DNA methyltransferase, which is associated with a larger survival benefit (Stupp et al., 2005; Stupp et al., 2009; van Genugten et al., 2010). When the achieved AUC is satisfactory and thus treatment effectiveness is fully understood, that is, when all stratification and confounding variables are known, the model can be used for accurate patient-level effectiveness predictions.

### Significance of the Proposed Methodology for RWD

In this temozolomide-glioblastoma case, the CT was potentially useful for exploring covariate interdependencies and confounders of individual treatment responses. With this, the importance of factors yet unknown to previously conducted clinical research, such as phenotypical or genotypical variations, can easily be integrated and tested for their effects using this technique. Therefore, CTs may be valuable in terms of discovering variations in patient-level effectiveness of medicines, which might not be discovered otherwise. This confirms recent literature discussing the promise of machine learning techniques in pharmaceutical innovation and decision making (Reps et al., 2018; Beam and Kohane, 2019; Rajkomar et al., 2019). Therefore, we argue that RWE-based machine learning analysis can be used in exploratory treatment effectiveness studies (Berger et al., 2017; Puranam et al., 2018) for improving the understanding of TRR and the specification of treatment paths with a level of detail not previously achieved in pharmacoepidemiology studies of temozolomide. In practice, when considering cancers that are being treated following multiple sequences (e.g. first- to third-line treatments) with a range of different, possibly combined, interventions (as is the case for melanoma, colorectal, and breast cancer) in conjunction with a range of different diagnostic tools, the technique can also be useful for exploring and predicting optimal treatment sequences and therefore guide clinical decision making.

### Limitations of the Proposed Method

This study does not come without limitations. For the CT’s predictive accuracy, the quality of the RWD is very important. Within health care, data sources may be of low veracity, that is, they may contain incomplete, imprecise, or inconsistent data. Data cleaning is an important step to mitigate this problem. Also, data sources may capture a low variety of information. Here, no primary data on treatment response was available, which required the use of PS matching to estimate personal treatment effect. Also, the BCR does not dispose of genetic information.

Additionally, we must note that the TRR definition did not consider survival censoring, that is, the OS of both treated and control patients were assumed to be uncensored. Fortunately, in this study, censoring was rarely observed given the severity of the disease; only 1% of matched cohort patients (13 of 1063) and 7% of treated patients (211 of 3090) had censored OS, and the latter was only of importance if the SG was less than one month because these would potentially be wrongfully classified as non-responsive. In such cases, the use of semi-supervised machine learning methods, where treatment response as the target feature is missing when the OS of either matched treated patient and/or matched control patient is censored, may improve these results.

Lastly, the used matching technique does not control for unobserved variables and does not consider early patient death before start of treatment. In our case, the latter may be important because of short patients’ OS.

## Conclusions

Using machine learning, we showed an increased understanding of patient-level treatment responses and specification of individual treatment paths that were not be identified using cohort-oriented methods used in previous RCT studies. Through the iterative learning model, confounding factors can be identified to achieve the most optimal prediction model of patient-level effectiveness.

We believe that machine learning can be effective in the observational phase following “initial” licensing in an adaptive licensing approach, as suggested by Eichler et al. (2012), or in the pilot phase after licensing following Phase III pre-approval studies in the sequential study design suggested by Franklin et al. (2014). In both cases, machine learning can be used for exploratory treatment effectiveness studies where hypotheses are generated to further guide efficient designs of large-scale confirmatory observational trials, both in disease database and pragmatic RCTs.

The CT method was found to be the suitable for this case because of its high interpretability and capability to deal with covariate interdependencies. However, the CT is suitable up to a maximum level of complexity characterized by the number of baseline variables, amount of possible treatment pathways and their combinations, and extent of OS censoring. Thus, when considering medicinal products such as cetuximab or panitumumab for colorectal cancer, CTs become inadequate because more patients will have censored OS while receiving multiple and more combined treatments in different sequences depending on their genetic expression, resulting in a smaller sample-to-feature ratio. As a result, methods should account for label uncertainty, for example, by including the likelihood of the treatment response measure. Further studies involving predictive data analytics used for real-world effectiveness exploration are needed to determine whether more advanced techniques within machine learning should be considered to deal with the higher complexity in these cases. These methods include probability-based Bayesian classification, support vector machines, and neural networks conducted through supervised or semi-supervised learning.

## Data Availability Statement

The data sets that support the findings of this study are available from the Belgian Cancer Registry but restrictions apply to the availability of these data, which were used under license for the current study, and so are not publicly available. Data are however available from the authors upon reasonable request and with permission of the Belgian Cancer Registry.

## Author Contributions

TG and WD participated in the design of the research, interpretation of the results and writing the manuscript. TG performed the data retrieval, statistical analysis and made substantial contributions to the writing of the manuscript. ND provided the materials and assisted in data cleaning. TG, WD, IH, and ND read and amended the manuscript and approved the final version for publication.

## Funding

This work was supported by the Vlerick Business School Academic Research Fund. The funding agreement ensured the authors’ independence in designing the study, interpreting the data, and publishing the report.

## Conflict of Interest

The authors declare that the research was conducted in the absence of any commercial or financial relationships that could be construed as a potential conflict of interest.

## Supplementary Material

The Supplementary Material for this article can be found onlineat: https://www.frontiersin.org/articles/10.3389/fphar.2019.01665/full#supplementary-material
