# Supplementary material for: Patient-Level Effectiveness Prediction Modeling for Glioblastoma Using Classification Trees
Source: Front Pharmacol. 2020 Jan 31;10:1665. doi: 10.3389/fphar.2019.01665 (PMC7025482; doi:10.3389/fphar.2019.01665)
Supplement: Supplementary file 1 [file DataSheet_1.docx]

**Appendix**

***Appendix I. Pseudo-code for decision tree analysis***

| **Exploratory learning algorithm** | |
| --- | --- |
| 1.  2.  3.  4.  5.  6.  7.  8.  9.  10.  11.  12.  13.  14.  15.  16.  17.  18.  19.  20.  21.  22.  23.  24.    25.  26. | ***Phase 0: Initialization***  Load DATA  T ← DATA[treatment group]  C ← DATA [cohort group]  ***Phase 1: Data Labelling***  ***Phase 1.1: Variable selection***  Select variables (p < 0.05) using logistic regression on one year survivability in DATA  ***Phase 1.2: Matching algorithm*** $\forall i\in DATA c$alculate ${PS}_{i}=Pr(Z_{i}=1\vert\boldsymbol{X}_{i})$ using selected variables from phase 1.1  **foreach** treated patient $i\in T$  select N nearest control patients $j\in C$ based on $PS$  match *i* and *j* → $j\in C(i)$  ***Phase 1.3: Target Labelling***  **foreach** treated patient $i\in T$ **with** $j\in C(i)$ matched  ${SG}_{i}\leftarrow{OS}_{i}-\frac{1}{N}\sum{OS}_{j}$  **if** ${SG}_{i}$ > λ months **then** response*_i_* = 1  **else** response*_i_* = 0  **end for**  ***Phase 2: Decision Tree Classification***  ***Phase 2.1: Build CART model***  T^train^ ← T[select 80% of data set]  T^test^ ← T[select the other 20% of data set]  train CART using T^train^ and 10-fold cross validation  ***Phase 2.2: ROC analysis***  Calculate ROC using T^test^  Calculate AUC of ROC  ***Phase 3: Iteration***  **while** AUC < threshold  ***Phase 3.1: Variable inclusion***  Inspect unobserved confounding variables: Go back to phase 2  ***Phase 3.2: Adapt target label***  Go back to phase 1 |

**Supplementary materials**

***I. Initialization phase: defining binary response***

Observational data were labelled by introducing a definition for treatment response, based on the gain in overall survival (OS) for each patient and estimated using nearest neighbour Propensity Score (PS) matching. In the following, the methodology for variable selection for the PS regression is explained. After this, details of the nearest neighbour PS matching technique are given.

***I.I Variable selection for nearest neighbour PS matching***

Observed variables X are selected based on their significant impact on the survival time, because this variable selection approach is associated with better PS estimations. Using a logistic regression model with the one-year survivability (=0 when OS < one year, = 1when OS ≥ one year) as binary dependent variable (Table A1), the variables significantly affecting survival were identified. These variables are age, RT and chemotherapeutic treatment (p-value < 0.001) and WHO performance score (p-value < 0.01) (Table A2).

| Category | Amount | Percentage |
| --- | --- | --- |
| 0 (survived less than one year) | 2801 | 61.70 |
| 1 (survived one year or more) | 1727 | 38.30 |

***Table A.1 Distribution of the dependent variable.***

| Variable | Coefficient | SE | p value |  |
| --- | --- | --- | --- | --- |
| *θ_0_* | -0.0526 | 0.5915 | 0.92914 |  |
| Age | -0.0499 | 0.0030 | <2e-16 | * |
| Sex | -0.1601 | 0.0728 | 0.02795 |  |
| Differentiation grade | -0.0676 | 0.1097 | 0.53761 |  |
| Total tumours | 0.0272 | 0.1115 | 0.80725 |  |
| WHO score | -0.1670 | 0.0551 | 0.00245 | * |
| Surgical status | 0.2072 | 0.3135 | 0.50869 |  |
| Radiotherapy status | 2.7329 | 0.1701 | <2e-16 | * |
| Chemotherapy status | 1.0204 | 0.0869 | <2e-16 | * |

***Table A.2 Outcome of logistic regression using one-year survivability as dependent variable. (*Significant)***

***I.II Estimation of the PS***

The PS is defined as $Pr(Z_{i}=1|X_{i})$, that is, the likelihood of a patient being treated with temozolomide (Z = 1) conditional on observed covariates X. The PS value is estimated using the following logit model:

$$log\left( \frac{PS}{1-PS} \right)=log\left( \frac{Pr(Z|X)}{1-Pr(Z|X)} \right)=\theta_{0}+\theta X$$

The outcome and distribution of the PS logit model is given in Table A3 and Figure A1.

| Variable | Coefficient | SE | p value |  |
| --- | --- | --- | --- | --- |
| *θ_0_* | 0.5995 | 0.0300 | <2e-16 | * |
| Age | -0.0067 | 0.0004 | <2e-16 | * |
| WHO score | -0.0014 | 0.0068 | 0.833 |  |
| Radiotherapy status | 0.6406 | 0.0127 | <2e-16 | * |
| Chemotherapy status | 0.0960 | 0.0129 | 1.42e-13 | * |

***Table A.3 Outcome of logit regression using PS as dependent variable. (*Significant)***


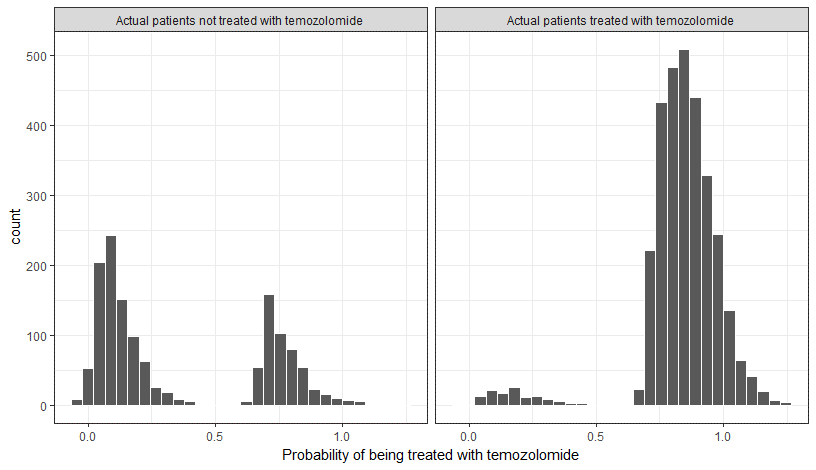


***Figure A.1 Distribution of propensity scores for the entire observational data set for patients in the control group (left) and treated group (right).***

***I.III Nearest neighbour PS matching***

Let $T$ and $C$ be the set of treated (Z=1) and control (Z=0) patients respectively. The gain in OS for each treated patient is given by ${{SG}_{i}}^{T}={{OS}_{i}}^{T}-\frac{1}{10}\sum_{j=1}^{10} {{OS}_{j}}^{C}$, with ${{OS}_{i}}^{T}$ defined as the observed OS of the treated patient $i\in T$and ${{OS}_{j}}^{C}$ defined as the observed OS of the control patient $j\in C$matched to $i$. Matching with replacement was used so that a control patient could be matched multiple times to a patient from the temozolomide group to prevent unmatched treatment units. Here, each temozolomide treated patient is matched to *k =10* control patients given $dist\left( {PS}_{i}^{T},{PS}_{j}^{C} \right)$ is minimal. Based on the ESMO MCB scale by Cherny (2015) (according to the form for non-curative oncological treatments with a median OS for standard treatment larger than one year and MCB grade of 3, as assigned to temozolomide from its efficacy results), treatment response (TR) was defined as:

${{TR}_{i}}^{T}= \left\{ \begin{aligned} 1 \mathrm{if}{{SG}_{i}}^{T}\geq1 \mathrm{monhts} \\ 0 \mathrm{else} \end{aligned} \right.$

***II. Machine learning phase: Decision Tree***

After the data labelling process, the DT was trained and validated ( ‘rpart’ package in R automatically performs 10-fold cross validation, and thus no separate training and validation set should be constructed). The dataset was divided into a training set (80% of the data set) and a test set (20% of the data set). Output of the model is shown in Table A4, together with the classification probabilities for response and non-response at each node.

| **Node** | **N** | **N corr** | **N loss** | **DT prediction** | **Probability of response** | **Probability of non-response** |
| --- | --- | --- | --- | --- | --- | --- |
| 1) Root | 2472 | 1286 | 1186 | 1 | 0.52 | 0.48 |
| 2) Age < 52 years * | 630 | 416 | 214 | 0 | 0.34 | 0.66 |
| 3) Age ≥ 52 years | 1842 | 1072 | 770 | 1 | 0.58 | 0.42 |
| 6) Chemo = yes | 432 | 250 | 182 | 0 | 0,42 | 0.58 |
| 12) Age < 63 years * | 210 | 176 | 34 | 0 | 0.16 | 0.84 |
| 13) Age ≥ 63 years * | 222 | 148 | 74 | 1 | 0.66 | 0.34 |
| 7) Chemo = no | 1410 | 890 | 520 | 1 | 0.63 | 0.37 |
| 14) Age > 61 years | 945 | 525 | 420 | 1 | 0,56 | 0,44 |
| 28) Age < 63 years * | 65 | 53 | 12 | 0 | 0.18 | 0.82 |
| 29) Age ≥ 63 years * | 880 | 513 | 367 | 1 | 0.58 | 0.42 |
| 15) Age ≤ 61 years * | 465 | 365 | 101 | 1 | 0.78 | 0.23 |

***Table A.4*** *Summary of the DT model after training and validation. DT stratification variables include age, RPA class, and chemotherapeutic (Chemo) and radiotherapeutic (RT) status. Note: N = number of patients in the node, N corr = number of true-positives/true-negatives, N loss = number of false-positives/false-negatives, DT prediction 0 indicates non-response, DT prediction 1 indicates response, * Terminal node.*

The model was then evaluated by estimating the AUC of the ROC of the test data. The ROC maps model sensitivity and specificity for various probability thresholds at which the DT predicts a response. Sensitivity and 1-specificity are defined as the true-positive rate (TPR) and false-positive rate (FPR), respectively, given by:

$$TPR= \frac{TP}{TP+FN}, FPR=\frac{FP}{FP+TN} ,$$

with true-positives (TP) and false-positives (FP) defined as the number of treated patients from the test set for which the DT predicts response correctly and incorrectly, respectively, with respect to the labelled value. True-negatives (TN) and false-negatives (FN) are the number of treated patients for which the DT predicts non-response correctly and incorrectly, respectively (Table A5). Hence, sensitivity describes the ability of the DT to correctly identify the patient who would actually respond to the treatment, while specificity describes the ability to correctly identify the patients who would not respond to the treatment.

|  |  | **True value** | |
| --- | --- | --- | --- |
|  |  | **Response** | **Non-response** |
| **DT prediction** | **Response** | True-positive | False-positive |
|  | **Non-response** | False-negative | True-negative |

***Table A.5 Contingency table of correct and incorrect DT classification predictions with respect to the target value labelled using nearest neighbour PS matching (true value).***

The ROC of the DT model given in Table A4 is depicted in Figure A2. While a random classifier corresponds to the diagonal line in the ROC (AUC is 50%), an optimal prediction corresponds with a curve passing close to the upper left corner, representing 100% sensitivity (TPR = 1, no false-negatives) and 100% specificity (FPR = 0, no false-positives). The ROC for our DT model had an AUC of 66,50% corresponding to a sensitivity of 0.6850 and a specificity of 0.5114.


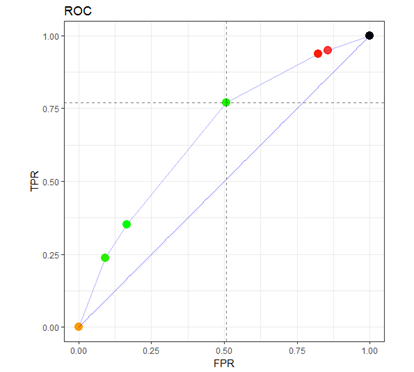


***Figure A.2*** ***ROC estimated using test data for the DT model represented in Figure 3.***

Note that the DT results strongly depend on early assumptions; for example, changing the target feature by modifying the response threshold from 1 to 3 months leads to a different DT structure with an AUC of 60%.
